# Carbapenemase-producing Enterobacteriaceae in Mexico: report of seven non-clonal cases in a pediatric hospital

**DOI:** 10.1186/s12866-018-1166-z

**Published:** 2018-04-19

**Authors:** Alejandra Aquino-Andrade, Jocelin Merida-Vieyra, Eduardo Arias de la Garza, Patricia Arzate-Barbosa, Agustín De Colsa Ranero

**Affiliations:** 1 Molecular Microbiology Laboratory, Instituto Nacional de Pediatría, Insurgentes Sur 3700-C, Insurgentes Cuicuilco, ZC, 04530 Coyoacán Mexico City, Mexico; 2 Pediatric Infectious Disease Department, Instituto Nacional de Pediatria, Mexico City, Mexico; 3 Clinical Bacteriology Laboratory, Instituto Nacional de Pediatria, Mexico City, Mexico

**Keywords:** Carbapenemase-producing Enterobacteriaceae, Pediatrics, Mexico, NDM-1, KPC-2, OXA-48, OXA-232

## Abstract

**Background:**

Carbapenemases-producing Enterobacteriaceae (CPE) are a worldwide public health emergency. In Mexico, reports of CPE are limited, particularly in the pediatric population. Here, we describe the clinical, epidemiological, and molecular characteristics of seven consecutive cases in a third-level pediatric hospital in Mexico City over a four-month period during 2016.

**Results:**

The Enterobacteriaceae identified were three *Escherichia coli* strains (producing OXA-232, NDM-1 and KPC-2), two *Klebsiella pneumoniae* strains (producing KPC-2 and NDM-1), one *Klebsiella oxytoca* strain producing OXA-48 and one *Enterobacter cloacae* strain producing NDM-1. The majority of patients had underlying disesases, three were immunocompromised, and three had infections involved the skin and soft tissues. Half patients died as a result of CPE infection.

**Conclusions:**

This study represents the first report of *E. coli* ST131-O25b clone producing NDM-1 in Latin America. In addition, this study is the first finding of *K. oxytoca* producing OXA-48 and *E. coli* producing OXA-232 in Mexican pediatric patients.

## Background

Currently, antimicrobial resistance is considered a global public health problem of highest priority, and the emergence of CPE is increasing. This situation is especially alarming due to the ease of dispersion of these resistance mechanisms, the difficulty in choosing adequate antimicrobial therapy, and the increase in mortality and hospital stay lengths caused by infections with these pathogens [1]. The mortality of patients infected with CPE varies from 26% to 44% [2] and can reach as high as 85% in patients with CPE bloodstream infections [3].

Carbapenem resistance in Enterobacteriaceae is largely mediated by the presence of enzymes known as carbapenemases, which have the capacity to inactivate beta-lactam antibiotics, including third- and fourth-generation cephalosporins and carbapenems. The most common carbapenemases in Enterobacteriaceae are the VIM, IMP, KPC, NDM and OXA types [4].

The frequency of CPE isolation varies among regions of the world. In the U.S., the CPE frequency from 1999 to 2012 was 0.08%, and species of *Enterobacter* were the most common isolates (0.57%) [5]. The Antimicrobial Surveillance Program SENTRY, carried out in 18 European countries from 2010 to 2013, evaluated 14,286 Enterobacteriaceae isolates and found that 2% were CPE, with a frequency varying from 0.1% (Ireland) to 17.3% (Poland). The most common CPE species were *K. pneumoniae* (86.4%) and *E. cloacae* (7.9%), and the most frequent carbapenemases found in this study were KPC 2/3 (85.4%), VIM (12.5%), and IMP-19 (2.1%) [6]. The equivalent program in Latin America, which included 11 countries during 2011–2014, revealed that 4.3% of isolates of Enterobacteriaceae were CPE, with the highest frequencies reported in Brazil (9%) and Argentina (6.3%). In Mexico, the reported frequencies of CPE were 0.7% [7]*.*

In the National Institute of Pediatrics (INP) we performed a retrospective analysis of Enterobacteriaceae isolates collected during February 2013–January 2015, based on antimicrobial susceptibility testing and molecular tests, and we only identified four *E. cloacae* isolates producing VIM-2.

In April 2016, the Pediatric Infectious Diseases Department detected a CPE in a patient with sepsis and neutropenic colitis. In the next months, six additional CPE were reported in the INP.

In this study, we describe the clinical, epidemiological and molecular data from a series of consecutive infection cases caused by seven CPE during a four-month period.

## Methods

### Study site

The INP is a public teaching-hospital with 243 beds, and is one of the largest pediatric reference centers in Mexico.

### Clinical aspects

Over a four-month period (April–July 2016), seven non-clonal Enterobacteriaceae isolates causing different clinical infections were obtained. The resistance profiles of these bacteria suggested that they were carbapenemases producers.

We reviewed the medical file from each patient to collect clinical and epidemiological data such as: demographic characteristics, underlying medical condition, previous antibiotic treatment, surgical procedure, mechanical ventilation, stay in pediatric intensive care unit (PICU), and number of clinical departments during hospitalization, among others. Acquisition of the different infections was determined to be healthcare-associated infections according to the definitions of the Centers for Disease Control and Prevention [8]. For this study, a case was defined as the appearance of at least one infection by a CPE that was clinically and microbiologically documented.

### Microbiological methods

The identification and susceptibility profiles of the isolates were performed using the Phoenix® BD system (Becton Dickinson, Sparks, MD, U.S.). The production of extended spectrum beta-lactamases (ESBL) and carbapenemases was confirmed phenotypically using the combined disk method and the CarbaNP test, respectively. We determined the minimum inhibitory concentration to colistin according to the Clinical Laboratory Standards Institute (CLSI) [9].

### Beta-lactamase typing

We extracted DNA from each isolate using the QIAamp® DNA Mini kit (QIAGEN, Hilden, Germany). We detected beta-lactamases by PCR amplification of *bla*_CTX-M-1,_ *bla*_CTX-M-2,_ *bla*_CTX-M-9,_ *bla*_SHV,_ *bla*_TEM,_ *bla*_LAT,_ *bla*_DHA,_ *bla*_VIM_, *bla*_IMP_, *bla*_NDM_, *bla*_KPC_ and *bla*_OXA-48_ genes using a GenAmp PCR System 9700 thermal cycler (Applied Biosystems Foster City, CA, USA). AmpliTaq Gold® 360 MasterMix (Applied Biosystems) was used for all reactions; the primers and amplification conditions were described previously [10–12]. The amplified fragments were purified using the QIAquick PCR purification kit (QIAGEN), and each product obtained was sequenced using a 3500 XL System (Applied Biosystems). We determined the beta-lactamase subtype using the BLAST bioinformatic tool.

### Multilocus sequence typing

Multilocus sequence typing (MLST) was performed on the *E. coli*, *K. pneumoniae*, and *E. cloacae* isolates [13–15]. We amplified *trpA, pabB* and *rfb* genes using conditions previously described, to detect the O25b-ST131 clone for all *E. coli* isolates [16].

## Results

### Bacterial isolates and detection of beta-lactamases

We identified three strains of *E. coli,* two of *K. pneumoniae*, one of *Klebsiella oxytoca*, and one of *E. cloacae*. All isolates were carbapenem resistant but showed differences in their susceptibility profiles to other antibiotic families, no resistance to colistin was observed in any isolate (Table 1). The confirmatory test for ESBL detection was positive for two isolates (C1 and C2). Coexistence of other beta-lactamases, including TEM-1, SHV-1 (non-ESBL), SHV-12, and CTX-M-15 (ESBL), was found in five isolates, two isolates with CTX-M-15 and NDM-1 were negative for ESBL test (Table 2). None isolate possessed genes encoding enzymes of the CTX-M-2, CTX-M-9, DHA, or LAT type.

**Table 1:** Resistance profile of carbapenemase-producing Enterobacteriaceae

Case	Species	MIC (mg/L)																				
		IMP	MEM	ERT	AMP	AMP/SULB	CFZ	CXM	FOX	CAZ	CRO	FEP	PTZ	AZT	CIP	LEV	GE	AK	TOB	SXT	TE	COL
1	*E. coli*	> 8	> 32	≥1	> 16	> 16/8	> 16	> 16	> 16	> 16	> 32	> 16	> 64/4	> 16	> 2	> 4	> 8	≤8	> 8	> 2/38	> 8	0.5
2	*K. pneumoniae*	8	8	≥1	IR	> 16/8	> 16	> 16	> 16	> 16	> 32	> 16	> 64/4	> 16	≤0.5	≤1	≤2	> 32	≤2	> 2/38	> 8	1.0
3	*E. coli*	4	4	≥1	> 16	> 16/8	> 16	> 16	> 16	> 16	> 32	> 16	> 64/4	> 16	≤0.5	≤1	> 8	≤8	8	≤0.5/9.5	> 8	0.5
	*K. pneumoniae*	≥8	32	≥1	IR	> 16/8	> 16	> 16	> 16	> 16	> 32	> 16	> 64/4	> 16	2	≤1	> 8	≤8	8	> 2/38	> 8	0.5
4	*K. oxytoca*	4	2	≥1	> 16	> 16/8	> 16	8	≤4	≤0.5	≤2	≤1	> 64/4	≤2	2	≤1	≤2	≤8	≤2	≤0.5/9.5	≤2	0.5
5	*E. coli*	> 8	16	≥1	> 16	> 16/8	> 16	> 16	> 16	> 16	> 32	> 16	> 64/4	> 16	> 2	> 4	> 8	≤8	> 8	> 2/38	> 8	0.5
6	*E. cloacae*	> 8	32	≥1	IR	IR	IR	IR	IR	> 16	> 32	> 16	> 64/4	> 16	> 2	> 4	> 8	> 32	> 8	> 2/38	> 8	0.5

*MIC* minimum inhibitory concentration, *IMP* imipenem, *MEM* meropenem, *ERT* ertapenem, *AMP/SULB* ampicillin/sulbactam, *CFZ* cefazolin, *CXM* cefuroxime, *FOX* cefoxitin, *CAZ* ceftazidime, *CTX* cefotaxime, *CRO* ceftriaxone, *FEP* cefepime, *PTZ* piperacillin/tazobactam, *AZT* Aztreonam, *CIP* ciprofloxacin, *LEV* levofloxacin, *GE* gentamicin, *TOB* tobramycin, *AK* amikacin, *SXT* trimethoprim sulfamethoxazole, *TE* tetracycline, *NIT* nitrofurantoin, *COL* colistin, *IR* intrinsic resistance

**Table 2:** Genotype characteristics of carbapenemase-producing Enterobacteriaceae

Case	Species	ESBL	Co-existing beta-lactamases	CarbaNP test	CBP	MLST
1	*E. coli*	+	CXT-M-15	–	OXA-232	ST2003
2	*K. pneumoniae*	+	SHV-12	+	NDM-1	ST76
3	*E. coli*	–	–	+	KPC-2	ST457
	*K. pneumoniae*	–	SHV-1^*^	+	KPC-2	ST5
4	*K. oxytoca*	–	–	–	OXA-48	ND
5	*E. coli*	–	CXT-M-15	+	NDM-1	ST131-O25b
6	*E. cloacae*	–	CXT-M-15, TEM-1 ^*^	+	NDM-1	ST182

*ESBL* phenotype test for the detection of extended-spectrum beta-lactamases, *CBP* carbapenemases, −: negative, +: positive, ^*^: non-ESBL, ND: not determined

### Phenotypic tests, carbapenemases detection and MLST

Five isolates were positive for the CarbaNP test. However, carbapenemase production was not detected using this technique in isolates producing OXA-type enzymes. Four carbapenemase types were detected: NDM-1, KPC-2, OXA-48 and OXA-232 in the seven isolates studied (Table 1). The sequence types (ST) of the *E. coli* isolates were ST2003 (C1), ST457 (C3), and ST131 (C5); *K. pneumoniae* isolates were ST5 (C3) and ST76 (C2), and *E. cloacae* ST182 (C6) (Table 2).

### Clinical and epidemiological characteristics

These isolates were obtained from six patients, one of whom had an infection with two isolates (C3), both producing KPC-2. With the exception of one female, all patients were male. The average age was 6.7 years (range 4 months - 16 years). Five patients had an underlying disease and three, were immunocompromised (C1, C4 and C5). Three patients presented skin and soft tissue infections (C2, C3 and C6). All deaths occurred in patients who CPE was isolated in blood. All of them had an intra-abdominal source of infection and secondary sepsis (Table 3).

**Table 3:** Characteristics of patients with carbapenemase-producing Enterobacteriaceae

Data	Cases					
	1	2	3	4	5	6
	*E. coli*	*K. pneumoniae*	*E. coli* *K. pneumoniae*	*K. oxytoca*	*E. coli*	*E. cloacae*
	OXA-232	NDM-1	KPC-2	OXA-48	NDM-1	NDM-1
Base diagnosis	AML-M2	Intestinal malrotation	KTWS	preB-ALL	Trisomy 21, Fallot Tetralogy	Complicated varicella
Immuno-compromised	Yes	No	No	Yes	Yes	No
Infection	Sepsis, Neutropenic colitis	Necrotizing fasciitis, SSI	Cellulitis, SSI	Abdominal sepsis	UTI	Necrotizing fasciitis
Sample	Blood, peritoneal liquid	Blood, wound drainage	Surgical wound drainage	Blood	Urine	Wound drainage
ID time^a^	10	8	22	39	157	9
Other hospital	No	Yes	No	No	No	Yes
PICU	Yes	Yes	Yes	Yes	Yes	No
MV	Yes	Yes	Yes	Yes	Yes	No
Surgery	No	Yes	Yes	Yes	Yes	Yes
CVC	Yes	Yes	Yes	Yes	Yes	No
TPN	No	Yes	Yes	Yes	Yes	No
Previous AB						
3GC (days)	No	Yes (2)	Yes (23)	Yes (3)^c^	Yes (7)	Yes (3)
4CG (days)	Yes (1)	No	No	Yes (8)^c^	No	No
Carbapenems	Yes (8)	Yes (18)	Yes (42)^b^	Yes (26)^b^	Yes (37)	Yes (29)^b^
Colistin	No	No	No	No	Yes	No
Definitive Tx	COL+MEM	MEM + LEV	MEM + AK	MEM + PTZ	NIT	COL+MEM
LOS (days)	11	20	44	40	197	35
ND	2	2	3	3	3	2
DI	PICU	Surgery	PIDD	PIDD	PIDD	PIDD
Evolution	Deceased	Deceased	Alive	Deceased	Alive	Alive

*Dx* diagnosis, *AML-M2* acute myeloid leukemia M2, *KTWS* Klippel-Trenaunay-Weber syndrome, *preB-ALL* pre-B acute lymphoblastic leukemia, *SSI* surgical site infection, *UTI* urinary tract infection, *PICU* pediatric intensive care unit, *MV* mechanical ventilation, *CVC* central venous catheter, *TPN* total parenteral nutrition, *AB* antibiotics, *3GC* third generation cephalosporins, *Tx* treatment, *LOS* length of in-hospital stay, *COL* colistin, *MEM* meropenem, *AK* amikacin, *PTZ* piperacillin/tazobactam, *NIT* nitrofurantoin, *ND* number of clinical departments during hospitalization, *DI* Clinical department in which the isolate was obtained, *PIDD* Pediatric Infectious Diseases Department, ^a^Days since admission to identification of the organism, ^b^Isolation during meropenem treatment, ^c^Escalating regimen

Before sampling for CPE screening, all patients had been hospitalized with an average of 41 days (range 8 to 157 days); therefore, all infections were considered to be healthcare-associated. Only two patients were admitted to an institution other than INP in the 30 days prior to infection (C2 and C6). Five patients required admission to the pediatric intensive care unit (PICU), mechanical ventilation, and central venous catheter placement, whereas four patients received total parenteral nutrition. Five patients had surgery prior to CPE isolation (Table 3).

Regarding prior use of antimicrobials, all patients received third or fourth generation cephalosporins, whereas all patients were treated with carbapenems. CPE were isolated in one patient (C6) while receiving meropenem.

Only two patients (C1 and C6) received treatment with colistin days prior to the isolation of CPE (Table 3).

## Discussion

In this report, we describe the consecutive emergence of seven CPE isolates in a third-level pediatric hospital: three *E. coli* producing NDM-1, KPC-2 and OXA-232*,* two *K. pneumoniae* producing KPC-2 and NDM-1, one *K. oxytoca* producing OXA-48, and one *E. cloacae* producing NDM-1. These pathogens with these carbapenemases appear for the first time in our institution. Previous to this report, we did not have an active screening to detect colonizing patients. In this study, all infections were considered primary cases. None patient had secondary cases due to the control measures that were taken, such as strictly supervised hand washing, staff training, restriction of personnel in contact with the patients, isolation of the patients in private rooms, exhaustive cleaning and decontamination of physical areas, and alerts on their conditions in the medical records of the discharged patients for future admissions.

Worldwide, *K. pneumoniae, E. coli* and *Enterobacter* spp. are the most frequent species of Enterobacteriaceae producing carbapenemases [6, 7]. To date, *K. oxytoca* has rarely been associated with carbapenemase production [17]. In Mexico, *K. oxytoca* has been related to VIM-2 production [18]; in other Latin American countries, production of other carbapenemases, including KPC-2 [19], IMP-4 [20], and VIM-4, have also been reported [21].

Descriptions of CPE with KPC-2 in Mexico are limited. From 2010 to 2014, KPC-2 and KPC-3-producing isolates of *K. pneumoniae* were reported [22, 23]. In one patient, we found two isolates that produce KPC-2, one *K. pneumoniae* strain and another *E. coli* strain (C3)*.* This study is the first report of KPC-2-producing *E. coli* in pediatric patients in Mexico; this isolate belongs to ST457. In Australia, *E. coli* ST457 has been described with CMY-1 and CTX-M-15, isolated from dog feces and extra-intestinal infections [24] and in Korea with OXA-232 in healthy individuals colonized [25].

The first report of NDM-1 in Mexico was registered in 2012, and the first cases were associated with an outbreak of a *Providencia rettgeri* clone [26]. In Mexico, *E. coli* (ST617)*, E. cloacae* (ST182)*,* and *K. pneumoniae* (ST22) isolates from adult patients [27] and *K. pneumoniae* (ST22) isolates from pediatric patients [28] have also been found to produce NDM-1. In the present series, NDM-1 was the enzyme most commonly found. This study is the first report of *E. coli* (C5) and *E. cloacae* (C6) producing NDM-1 in pediatric patients in Mexico.

For C5, the isolation of NDM-1-producing *E. coli* belonging to clone ST131-O25b was obtained from a urinary tract infection (UTI). The ST131 clone is considered the most predominant pathogenic lineage of this species in extra-intestinal infections and is associated with resistance to fluoroquinolones and dissemination of the beta-lactamase CTX-M [29]. There are reports in Mexico of *E. coli* ST131-O25b causing UTI healthcare-associated and community acquired; these infections have been associated with CTX-M-15, but not NDM-1 [30]. NDM production by *E. coli* ST131 is rare but has been reported in clinical and environmental isolations in countries such as India, Vietnam, Serbia, Philippines [31] and U.S. [32, 33]. This is the first report of NDM-1-producing *E. coli* ST131-O25b in Latin America.

Patient C6, who was infected with NDM-1-producing *E. cloacae*, received colistin for the treatment of fasciitis and presented a favorable therapeutic outcome. This isolate belonged to ST182, this clone has been described in our country recently involved in a hospital outbreak during 2014–2015 [34].

We identified an OXA-48-producing *K. oxytoca* isolate with susceptibility to third- and fourth-generation cephalosporins but resistance to carbapenems. This pattern is characteristic of isolates producing this enzyme. Moreover, the coexistence of ESBL in the same isolate may lead to the suspicion of another type of carbapenemase [35]. Using the CarbaNP test, we did not detect carbapenemase activity in this isolate, as was previously described; therefore, this test is not recommended for the detection of the OXA-type enzyme phenotype [36]. Five isolates of OXA-48-producing *K. oxytoca* were reported in 2010 in Turkey [37]; however, in contrast to the strain isolated from C4, these strains produce other ESBL, including SHV, TEM, CTX-M, and VEB. One OXA-48-producing *K. oxytoca* isolate was reported in Israel with susceptibility to ceftazidime, ceftriaxone, gentamicin and meropenem but resistance to imipenem and ertapenem [38].

The first report of OXA-48 enzymes produced by *E. coli* in our country was described in a cohort of patients at risk of being CPE fecal carriers, and three *K. pneumoniae* and 13 *E. coli* isolates producing OXA-232 alone or in combination with other SHV and CTX-M-15 type beta-lactamases were obtained from this cohort [39]. Later, the same group reported that the most common carbapenemase in their hospital was OXA-232, mainly in *E. coli* and *K. pneumoniae;* infections by these microorganisms were associated with prior use of beta-lactams with beta-lactamase inhibitors and third-generation cephalosporins [40]. The *E. coli* with OXA-232 isolate (C1) belongs to ST2003 and additionally produces CTX-M-15. Although this ST has been associated with the production of other enzymes, such as KPC-2 and CTX-M-55 [41], there are no reports of OXA-232 production. The OXA-232 enzyme has also been found in *E. coli* ST457 and ST131 [25] and coexists in *E. coli* and *K. pneumoniae* isolates that produce other carbapenemases, such as NDM-1 [42, 43].

The two patients who suffered from CPE infections with OXA-type enzymes had leukemia as an underlying illness, the origin of these infections was abdominal, and both patients died (Table 3). The mortality reported by Enterobacteriaceae producing OXA-48-like enzymes reaches 50% [44]. This study represents the first report of OXA-48-producing *K. oxytoca* and OXA-232-producing *E. coli* in Mexican pediatric patients. Performing MLST for *K. oxytoca* was not possible.

The presence of other beta-lactamase enzymes was commonly reported in CPE, notably non-ESBL TEM and SHV, CTX-M-15, SHV-12, CMY and DHA subtypes, and sometimes other carbapenemases, such as VIM and IMP [16, 45–47]. We detected non-ESBL enzymes (TEM-1 and SHV-1) as well as ESBL (CTX-M-15 and SHV-12) in four isolates, but none produced enzymes of the CMY and DHA types or the other CTX-M subtypes. CTX-M-15 has also been found in CPE that produce NMD-1 [27]. However, CTX-M-15 and SHV-12 are the most commonly detected ESBL in other third-level hospitals in Mexico [30]. In two isolates, *E. coli* and *E. cloacae* with NDM-1 (case 5 and 6, respectively) we found CTX-M-15, but we could not phenotypically detect the production of ESBL, according to the literature this can be explained because the NDM type enzymes are not inhibited by clavulanic acid, which can intervene in the interpretation of the ESBL test, when NDM and CTX-M-15 co-exist in the same isolate [48]. A variety of risk factors have been considered in the acquisition of CPE, such as prior, recurrent, or prolonged hospitalization, the use of antimicrobials, immunosuppression, the presence of central venous catheters, intensive care unit (ICU) admission and recent surgery [49]. The majority of patients in this series had these risk factors.

The mortality in this study was high, because half patients died. This finding is similar to other reports in the literature; the mortality of patients with CPE infection reached 44% [2]. In our series, all patients with documented CPE with bloodstream infection died. Mortality can reach 85% in cases of bloodstream infection [3]. All cases were controlled, and no secondary cases appeared. The emergence of these CPE isolates was an institutional alarm because all cases were healthcare-associated infections; because we do not have a surveillance program for the detection of CPE carriers in the INP. Four patients among six had recurrent or prolonged hospitalization in our hospital, considered a risk factor. However, two cases (C2 and C6) were patients who came from other hospitals and in whom an infection was detected earlier (8 and 9 days, respectively). Therefore, these patients may have been colonized prior to admission at our institution. The role of CPE colonization at the intestinal level is well documented and allows cross-transmission and dissemination in healthcare institutions [49, 50]. CPE as the cause of outbreaks is a growing problem that is reported at a global level. Therefore, establishing screening programs for the early identification of these pathogens through rectal swabs of these patients is important [50], as is the implementation of prevention and control measures to avoid dissemination of these pathogens [49, 50].

## Conclusions

This study reports the clinical, epidemiological and molecular characteristics of seven consecutive CPE cases. We report the finding of Enterobacteriaceae isolates producing carbapenemases not previously detected in Mexican pediatric patients. Finally, this is the first report of an NDM-1-producing *E. coli* ST131-O25b clone in Latin America.

The monitoring, surveillance, and control of CPE should be reinforced due to the ease of resistance cross-transmission among these pathogens, the possibility of dissemination, and the limited therapeutic possibilities.

## Abbreviations

3GC: third generation cephalosporins
AB: antibiotics
AK: amikacin
AML-M2: acute myeloid leukemia M2
AMP/SULB: ampicillin/sulbactam
AZT: aztreonam
BLAST: Basic Local Alignment Serach Tool
CAZ: ceftazidime
CBP: carbapenemases
CFZ: cefazolin
CIP: ciprofloxacin
CLSI: Clinical Laboratory Standards Institute
CMY-1: cephamycin AmpC beta-lactamase
COL: colistin
CPE: Carbapenemase-producing Enterobacteriaceae
CRO: ceftriaxone
CTX: cefotaxime
CTX-M: Cefotaximase–München
CVC: central venous catheter
CXM: cefuroxime
DHA: Dhahran Hospital, Saudi Arabia AmpC beta-lactamase
DI: Clinical department in which the isolate was obtained
Dx: diagnosis
ERT: ertapenem
ESBL: extended spectrum beta-lactamases
FEP: cefepime
FOX: cefoxitin
GE: gentamicin
ICU: intensive care unit
IMP: imipenem
IMP: imipenemase
INP: National Institute of Pediatrics
IR: intrinsic resistance
KPC-2: *Klebsiella pneumoniae* carbapenemase
KTWS: Klippel-Trenaunay-Weber syndrome
LAT: latamoxef AmpC beta-lactamase
LEV: levofloxacin
LOS: length of in-hospital stay
MEM: meropenem
MIC: minimum inhibitory concentration
MLST: Multilocus Sequence Typing
MV: mechanical ventilation
ND: not determined
ND: number of clinical departments during hospitalization
NDM-1: New Delhi metallo-β-lactamase
NIT: nitrofurantoin
OXA-232: oxacillinase-232
OXA-48: oxacillinase-48
PICU: pediatric intensive care unit
PIDD: Pediatric Infectious Diseases Department
preB-ALL: pre-B acute lymphoblastic leukemia
PTZ: piperacillin/tazobactam
SHV: Sulfhydryl variable beta-lactamaase
SSI: surgical site infection
ST: sequence types
SXT: trimethoprim sulfamethoxazole
TE: tetracycline
TEM: Temoneira beta-lactamase
TOB: tobramycin
TPN: total parenteral nutrition
Tx: treatment
UTI: urinary tract infection
VEB: Vietnamese extended-spectrum beta-lactamases
VIM: Verona integron-encoded metallo-β-lactamase
